# Cowper’s Syringocele: A Literature Review

**DOI:** 10.7759/cureus.32583

**Published:** 2022-12-16

**Authors:** Saryia Javed, M Hasaan Shahid, Islam Omar, Sapna Gupta, Anwar Zeb Khan, Adeel Kaiser

**Affiliations:** 1 General Surgery, Queen Elizabeth University Hospital, Glasgow, GBR; 2 Surgery, Postgraduate Medical Institute, Lahore, PAK; 3 Surgery, Northern Health and Social Care Trust, Antrim, GBR; 4 General Surgery, University Hospital of Wales, Cardiff, GBR; 5 Surgery, Lahore General Hospital, Lahore, PAK

**Keywords:** syringocele classfication, obstructive urinary symptoms, pediatric population, adolescent, cystic dilatation, cowper's gland, cowper's syringocele

## Abstract

Cowper’s syringocele is a rare but underdiagnosed cystic dilatation of the main ducts of Cowper’s gland. It is becoming more widely known in the adult population. Recent research proposes that syringoceles should be categorized according to the intraductal pressures causing ductal dilatation from mild to gross ultimately involving the gland itself. Although there may be some overlap in the clinical manifestations of different syringoceles, mildly dilated ducts are frequently asymptomatic. Moreover, moderate to gross duct dilatations can manifest as lower urinary tract symptoms (LUTS) or obstructive symptoms. A valid differential diagnosis is essential because these symptoms can be found in a wide range of severe illnesses. Syringocele can be diagnosed by ultrasonography in combination with voiding retrograde/antegrade cystourethrogram (VCUG), nevertheless, other procedures like cystourethroscopy, CT scan, and MRI scans can be helpful. Initially, conservative surveillance is advised, but if necessary, endoscopic marsupialization or surgical excision is the preferred treatment modality to address persistent problems.

## Introduction and background

Cowper's glands are the two pea-sized glands named after William Cowper, who gave a detailed description of the gland in 1699, but Jean Mery, a French surgeon, first identified them in 1684. They are also known as bulbourethral glands [1-2]. Cowper's syringocele was discovered by Fenwick [3]. It is a rare but underdiagnosed condition of the male urogenital tract, which was conventionally considered to be evident in the pediatric age group but is now becoming more widely recognized in adults with the emergence of cases being reported in the literature lately. Even though they can compress the urethra and cause obstructive urinary tract symptoms, they are often not covered in depth in the literature. This article reviews the anatomy, physiology, classification, clinical manifestation, diagnosis, and management of Cowper's syringocele.

## Review

Anatomy and physiology

Cowper's gland develops from the urogenital tubercle, derived from the pericloacal mesoderm, for which androgens play a pivotal role [4]. They are located in the wide base of the external urethral sphincter muscles in the deep perineal pouch, posterolateral to the membranous urethra, and superior to the perineal membrane [5-6]. The Cowper's gland ducts open into the bulbous urethra in the superficial perineal pouch by penetrating the perineal membrane [7]. They are multilobular glands that secrete alkaline mucinous secretions containing glycoproteins into the penile urethra right before sperm ejaculation. Its primary function is to lubricate the semen and urethra, along with balancing the pH of the urine residue [8].

Cowper’s syringocele

Syringocele was initially defined by Maizels et al. in 1983 to classify the variations of Cowper's gland duct dilatation. It is derived from the Greek words "syringo" which means tube and "cele" which means swelling. It is a cystic dilatation of the main ducts of the Cowper's gland [9]. It can be inherited or acquired. Congenital syringocele pathogenesis is yet unknown [8]. However, research in mice has provided evidence that an insignificant amount of transforming growth factor-beta 2 (TGF-β2 level) could lead to the formation of a syringocele [10]. Acquired causes may include trauma caused by prolonged urethral catheterization or post-inflammatory ductal blockage following infection [11].

Classification

The most typical classification proposed by Maizels et al. is as follows: (1) simple syringocele -- minimally dilated duct; (2) perforated syringocele -- a diverticulum-like patulous connection with the urethra; (3) imperforate syringocele -- a submucosal cyst-like dilated bulbous duct; and (4) ruptured syringocele -- the thin membrane left in the urethra following a ruptured dilated duct [12]. This classification is mainly radiological and not based on clinical symptoms; therefore, to reflect clinical presentation, other authors have suggested modifying this classification. Campobasso et al. in 1996 suggested a simpler classification after analysis of 15 consecutive cases with Cowper's syringocele thus dividing it into two categories: obstructing type with obstructive urinary symptoms, and non-obstructing syringocele with haematuria, urinary incontinence, fever, and/or urinary tract infection (UTI) [13]. Nevertheless, Bevers et al. recommended that syringoceles can be categorized into open and closed types based on how the ducts open into the urethra, for instance, open syringoceles have a continuous lumen between the urethra and the ducts manifesting as urinary incontinence in contrast to closed syringoceles causing obstructive urinary symptoms [14].

However, all three classifications preclude the more complex syringocele form involving the bulbourethral glands. The most extensive study to date on Cowper's syringocele done by Bugeja et al. described the classification based on the pressures inside the duct that better explain the symptoms and eventually suggest appropriate management. According to them, the Cowper's gland also becomes involved with the subtle dilatation of the duct. Small cysts with mildly dilated ducts are frequently asymptomatic incidental observations that rarely need treatment, but with the increased pressures, the ducts become progressively dilated, leading to urinary outflow obstruction symptoms requiring intervention via endoscopically or open methods, ultimately involving the Cowper's glands itself, requiring surgical removal for their tendency to cause potential problems such as a perineoscrotal abscess or fistula formation [15].

Symptoms

Cowper’s syringocele can be asymptomatic or present with a variety of symptoms. Mild to moderate duct dilatation or open syringocele can manifest as post-void incontinence, hematuria, urethral discharge, and UTI. In contrast, closed syringocele or large cysts with grossly dilated ducts can present with obstructive urinary symptoms, for instance, dysuria, weak urine stream, urinary retention, and perineal pain [15-17]. In the most severe cases, it can cause antenatal bilateral hydroureteronephrosis resulting in intrauterine/perinatal death and in the neonates, it can result in vesicoureteral reflux progressing to hydronephrosis and renal failure [13]. In children, it can manifest as a scrotal mass/abscess [16]. In adults, especially if the gland is involved, can cause perineoscrotal mass/abscess and chronically discharging perineal sinus/fistula [15, 17-18].

Differential diagnosis

Based on the imaging, the differential diagnosis includes an ectopic ureter, anterior urethral valves, or diverticulum. In addition, periurethral lesions such as abscesses and benign or malignant tumors can create a diagnostic conundrum as the imperforated syringocele may appear as an external bulge that can be detected on a urethral sonogram [19-20].

Diagnosis

Detailed history-taking, which includes the urological symptoms, can help with the diagnosis [21]. Transperineal or transrectal ultrasound can be used to detect the Cowper's glands' cystic lesions with the benefits of being affordable, non-invasive, and radiation-free. However, its drawbacks include the possibility of failure due to insufficient tissue penetration, especially in the penile bulb [20]. A 7.5 MHz linear high-definition ultrasonography (US) probe was utilized by Pavlica et al. to investigate five patients with a syringocele depicting a "double tube"-like the tubular image at the bulbous urethra running along with the urethra. It is easily distinguished from urethral stenosis. However, it can be mistaken for extremely rare congenital urethral duplication. Particularly in young patients, the US with a small-part probe is a viable imaging tool and substitute for traditional radiography [22]. Diagnostic testing typically involves a voiding antegrade or retrograde cystourethrogram which is an invasive technique requiring a urethral catheterization and radiation to the patient. However, it is a gold standard for diagnosing syringocele communication with the urethra [23]. Cystourethroscope, an invasive procedure, shows an imperforate syringocele as an extrinsic mass arising from the bulbar urethral floor, while a perforated syringocele would manifest as a pouch connecting to the bulbous urethra [24]. Further investigations, such as urodynamic studies and CT or MRI scans of the perineum, can be carried out if more information is required [21]. MRI also helps diagnose Cowper's syringocele by exhibiting a homogeneous cystic lesion with the advantage of being radiation-free non-invasive imaging with a broader field of vision. Nevertheless, it is much more expensive with little availability than the US or voiding cystourethrogram (VCUG) [20, 23].

Management

Asymptomatic syringocele can be managed conservatively. Even though many symptomatic patients ultimately require surgical intervention, a trial period of conservative management appears rational because spontaneous symptom resolution over time is not uncommon [17]. Bevers et al. have recorded several confirmed cases of syringocele in which the symptoms gradually subsided, and, in one adult, the syringocele spontaneously resolved [14]. The preferred solution for symptomatic syringocele in recent years has been endoscopic intervention via hook of the diathermy or cautery scissors [24-25]. A typical, efficient method of marsupialization for open and closed syringes is to unroof the cyst by disconnecting it from the urethra. With a maximum follow-up period of almost two years in the Bevers et al. case series, all four patients experienced complete resolution of the symptoms who underwent the deroofing procedure [14, 17]. The successful implication of the Holmium: Yttrium Aluminum Garnet (YAG) laser for syringocele intervention which Piedrahita and Palmer initially proposed, is now more widely practiced, and recent case reports depicted that there is no need for urethral catheterization after this treatment [9, 21, 23, 26]. Open methods, for instance, ligation of the Cowper's duct by transperineal approach, provide an alternative but are typically used after a failed unroofing attempt [27]. Another minimally invasive approach may also be beneficial, such as the laparoscopic marsupialization of Cowper's syringocele [28]. In the pediatric age group above procedures can be considered. However, patients presenting with incompetent spongiosum and extensive diverticulum can undergo diverticulectomy and newborns with severe reflux problems may require urinary diversion and vesicostomy [17].

## Conclusions

Cowper’s syringocele -- a cystic dilatation of the main ducts of the bulbourethral glands may be prevalent more in adults than considered previously as most cases are asymptomatic and thus are incidental findings. In contrast, others may be the source of the lower or upper urinary tract problems or misdiagnosed as urethral diverticula or periurethral lesions, which frequently manifest as severe symptoms requiring surgical intervention. A valid differential diagnosis is essential as these symptoms can be found in a wide range of severe illnesses. A detailed urological history and a series of urological investigations can help for the accurate diagnosis, of which voiding cystourethrogram has the utmost importance. Compared to the earlier classifications, it is more practical from a clinical standpoint to categorize the progressive cystic dilatation according to the build-up of intraductal pressures, ultimately incorporating the gland itself, necessitating surgical intervention. Endoscopic deroofing with holmium laser is the preferred treatment modality in adults, but an obligation excision may be necessary for the pediatric age group. Even if the diagnosis and treatment of syringocele have reasonable success rates, more comparative data are necessary to develop standardized procedures.

## References

[1] Sanders MA (2005). William Cowper and his decorated copperplate initials. Anat Rec.

[2] Kutia SA, Sataeva TP, Nikolaeva NG, Printseva NY, Moroz GA (2016). [The history of discovery of bulbourethral glands]. Urologiia.

[3] Fenwick EH (1896). Clinical lecture on retention cysts of Cowper's glands as a cause of chronic gleet, spasmodic stricture, organic stricture, extravasation of urine, and of so-called "False Passages" in the bulbous urethra. Br Med J.

[4] Haschek WM, Rousseaux CG, Wallig MA, Bolon B, Ochoa R Haschek and Rousseaux's Handbook of Toxicologic Pathology (3rd ed.).

[5] Tayib A, Mosli H, Atwa M (2003). Cowper's syringocele (cyst of the bulbar urethra): a case report and literature review. Ann Saudi Med.

[6] Muro S, Suriyut J, Akita K (2021). Anatomy of Cowper's gland in humans suggesting a secretion and emission mechanism facilitated by cooperation of striated and smooth muscles. Sci Rep.

[7] (2014). Atlas of Male Genitourethral Surgery: The Illustrated Guide. https://ebookcentral.proquest.com/lib/nhsscotland-ebooks/reader.action.

[8] Chughtai B, Sawas A, O'Malley RL, Naik RR, Ali Khan S, Pentyala S (2005). A neglected gland: a review of Cowper's gland. Int J Androl.

[9] Becerra MF, Smith N, Bhat A, Shah HN (2022). Endoscopic management of adolescent closed Cowper's gland syringocele with holmium: YAG laser. Asian J Urol.

[10] Dünker N, Aumüller G (2002). Transforming growth factor-beta 2 heterozygous mutant mice exhibit Cowper's gland hyperplasia and cystic dilations of the gland ducts (Cowper's syringoceles). J Anat.

[11] Brock WA, Kaplan GW (1979). Lesions of Cowper's glands in children. J Urol.

[12] Maizels M, Stephens FD, King LR, Firlit CF (1938). Cowpek’s syringocele: a classification of dilatations of Cowper’s gland duct based upon clinical characteristics of 8 boys. J Urol.

[13] Campobasso P, Schieven E, Fernandes EC (1996). Cowper's syringocele: an analysis of 15 consecutive cases. Arch Dis Child.

[14] Bevers RFM, Abbekerk EM, Boon TA (2000). Cowper's syringocele: symptoms, classification and treatment of an unappreciated problem. J Urol.

[15] Bugeja S, Frost A, Ivaz S, Dragova M, Andrich DE, Mundy AR (2020). Syringoceles of Cowper's ducts and glands in adult men. Asian J Androl.

[16] Saadat P, Borzi P, Patel B, Winkle D (2019). Urethral syringocele: unseen but existing. ANZ J Surg.

[17] Melquist J, Sharma V, Sciullo D, McCaffrey H, Khan SA (2010). Current diagnosis and management of syringocele: a review. Int Braz J Urol.

[18] Sharbaugh AJ, Yecies TS, Rusilko PJ, Dasyam AK, Turner RM (2018). Cowper's gland syringocele. Urology.

[19] Kumar J, Kumar A, Babu N, Gautam G, Seth A (2007). Cowper's syringocele in an adult. Abdom Imaging.

[20] Kickuth R, Laufer U, Pannek J, Kirchner TH, Herbe E, Kirchner J (2002). Cowper's syringocele: diagnosis based on MRI findings. Pediatr Radiol.

[21] Taskovska M, Hawlina S (2017). Cowper's syringocele in adolescent male: case report. J Endourol Case Rep.

[22] Pavlica P, Barozzi L, Stasi G, Viglietta G (1989). [Ultrasonography in syringocele of the male urethra (ultrasound-urethrography)]. Radiol Med.

[23] Matta I, Chalhoub K, Abou Zahr R, Ghazal G, Huyghe E, Nohra J (2019). A case of symptomatic Cowper's syringocele in an adult male: diagnosis and management. J Endourol Case Rep.

[24] Merchant SA, Amonkar PP, Patil JA (1997). Imperforate syringoceles of the bulbourethral duct: appearance on urethrography, sonography, and CT. Am J Roentgenol.

[25] Awad MA, Alwaal A, Harris CR, Zaid UB, Gaither TW, Osterberg EC, Breyer BN (2016). Transurethral unroofing of a symptomatic imperforate Cowper's syringocele in an adult male. Case Rep Urol.

[26] Piedrahita YK, Palmer JS (2006). Case report: Cowper's syringocele treated with Holmium:YAG laser. J Endourol.

[27] Santin BJ, Pewitt EB (2009). Cowper's duct ligation for treatment of dysuria associated with Cowper's syringocele treated previously with transurethral unroofing. Urology.

[28] Cerqueira M, Xambre L, Silva V (2004). Imperforate syringocele of the Cowper's glands laparoscopic treatment. Actas Urol Esp.

